# Surgery for Perforated Peptic Ulcer: Is Laparoscopy a New Paradigm?

**DOI:** 10.1155/2021/8828091

**Published:** 2021-05-12

**Authors:** André Pereira, Hugo Santos Sousa, Diana Gonçalves, Eduardo Lima da Costa, André Costa Pinho, Elisabete Barbosa, José Barbosa

**Affiliations:** ^1^General Surgery Department, São João University Medical Center, Porto, Portugal; ^2^Faculty of Medicine, University of Porto, Porto, Portugal; ^3^Obesity Integrated Responsibility Unit (CRI-O), Porto, Portugal

## Abstract

**Introduction:**

Laparoscopic repair of perforated peptic ulcer (PPU) remains controversial mainly due to its safety and applicability in critically ill patients. The aim of this study is to compare the outcomes of laparoscopy versus laparotomy in the treatment of PPU.

**Methods:**

Single-institutional, retrospective study of all patients submitted to surgical repair of PPU between 2012 and 2019.

**Results:**

During the study period, 169 patients underwent emergent surgery for PPU. A laparoscopic approach was tried in 60 patients and completely performed in 49 of them (conversion rate 18.3%). The open group was composed of 120 patients (included 11 conversions). Comparing the laparoscopic with the open group, there were significant differences in gender (male/female ratio 7.2/1 versus 2.2/1, respectively; *p*=0.009) and in the presence of sepsis criteria (12.2% versus 38.3%, respectively; *p*=0.001), while the Boey score showed no differences between the two groups. The operative time was longer in the laparoscopic group (median 100' versus 80', *p*=0.01). Laparoscopy was associated with few early postoperative complications (18.4% versus 41.7%, *p*=0.004), mortality (2.0% versus 14.2%; *p*=0.02), shorter hospital stay (median 6 versus 7 days, *p*=0.001), and earlier oral intake (median 3 versus 4 days, *p*=0.021).

**Conclusion:**

Laparoscopic repair of PPU may be considered the procedure of choice in patients without sepsis criteria if expertise and resources are available. This kind of approach is associated with a shorter length of hospital stay and earlier oral intake. In patients with sepsis criteria, more data are required to access the safety of laparoscopy in the treatment of PPU.

## 1. Introduction

Peptic Ulcer Disease (PUD) is a clinical condition that results from an imbalance between ulcerogenic factors and mucosal defence barriers of the stomach and duodenum.

Recent data show a heterogeneous incidence and prevalence of PUD worldwide [1]. However, almost all of the authors agree that both of them are decreasing especially due to the eradication of *Helicobacter pylori* and the use of Proton Pump Inhibitors (PPI) [2]. Over the past 3 decades, the median age of diagnosis increased from the 40 s to the 60 s; a previously male-predominant disease affects now both sexes equally and the ulcer location is now more frequent in the stomach than in the duodenum [3, 4].

Although the incidence of PUD is decreasing, the total number of PUD complications, such as bleeding and perforation, remains stable [3]. Peptic ulcer perforation is the second most frequent complication after bleeding but it represents the main indication for emergent surgery for PUD, with short-term mortality and morbidity as high as 30 and 50%, respectively [2, 5].

Some perforations may resolve spontaneously and these patients can be managed nonoperatively. The presence of sepsis, generalized peritonitis, or failed nonoperative management is an indication for emergent surgery [6, 7]. Suture of the perforation, with or without an omental patch, has been accepted as the ideal procedure for the majority of cases. Usually, this procedure is performed by laparotomy, but with the widespread of laparoscopic procedures and concomitant surgeon expertise, a minimally invasive approach is increasingly being preferred. Recent studies stated that laparoscopic repair of PPU is performed in 1/3 of the patients [8, 9].

The aim of this study is to assess the feasibility and safety of laparoscopic repair of PPU, even in patients with sepsis criteria, compared to the classic open approach.

## 2. Materials and Methods

All patients submitted to surgery for gastric or duodenal PPU between January 2012 and December 2019 in our tertiary hospital were identified from the internal electronic database (Sclinico®). Demographic and clinical data were retrieved. Patients treated nonoperatively and those with gastric or duodenal malignancies were excluded.

A *per-protocol* analysis was performed to compare patients submitted to laparoscopic repair of PPU (*n* = 49) with patients submitted to open repair of PPU (*n* = 120, including 11 patients with the conversion of laparoscopic procedures to open surgery). Several parameters were evaluated: demographic and clinical-pathological characteristics (sex, age, comorbidities, presence of sepsis/septic shock, Boey score, ulcer localization, and symptoms-to-surgery time interval), surgical procedure (suture versus resection; ulcer biopsy), and outcomes (operative time, suture dehiscence, reintervention, morbidity, mortality, Clavien-Dindo classification, resumption of oral intake, and length of hospital stay).

Analysis of the subgroup of patients who presented with sepsis/septic shock criteria at the moment of diagnosis (*n* = 52, 30.8%) was also performed.

### 2.1. Severity Scores

To assess the presence of sepsis or septic shock at the moment of diagnosis, the 3rd International Consensus Definitions for Sepsis and Septic Shock (Sepsis-3) [10] were used. In patients with PPU, sepsis was assumed if ≥ 2 points were present on the Sequential Organ Failure Assessment (SOFA) score; the septic shock was assumed if vasopressors were needed to maintain mean arterial pressure ≥65 mmHg and serum lactate level was ≥2 mmol/L in the absence of hypovolemia.

Boey score was evaluated based on three criteria: the presence of major comorbidities, preoperative systolic arterial pressure <90 mmHg, and duration of symptoms >24h [11].

### 2.2. Statistical Analysis

Chi-square test was used to compare categorical variables and the nonparametric Mann–Whitney U test for the continuous data analysis. *Odds ratios* were assumed by logistic regression and adjusted for sex and age. A *p* value < 0.05 was considered statistically significant. SPSS 24.0 for Mac (SPSS Inc., Chicago, IL) was used to perform all statistical analysis.

## 3. Results

Between January of 2012 and December of 2019, 169 patients were submitted to surgical repair of PPU. Of these patients, 125 (74%) were males and 44 (26%) females. The median age was 52 years (range 21–97). Previous medical comorbidities were present in 97 patients (57.4%). Clinical-pathological characteristics, surgical procedures, and outcomes are presented in Table 1.

Laparoscopic repair of PPU was attempted in 60 (35.5%) patients and 81.7% of these patients (49/60) completed surgical treatment by this kind of approach. There was a conversion rate of 18.3% (11/60). Causes of conversions were technical difficulties in 7 patients, perforation on the posterior gastric wall in 2 patients, perforation of the abdominal aorta with the Veress needle in one patient, and ventilatory intolerance to pneumoperitoneum in another patient.

Patients were divided into two groups: laparoscopy group (49 patients) and laparotomy group (120 patients). Those patients who required conversion were included in the open surgery group.

When both groups were compared, significant differences were observed in gender distribution (male : female ratio was 7.2 : 1 in the laparoscopy group and 2.2 : 1 in the laparotomy group, *p*=0.009) and in age (median age in the laparoscopy group was 48 years and in the laparotomy group was 53 years, *p*=0.002).

At diagnosis, 33 (19.5%) patients presented with sepsis and 19 (11.2%) patients with septic shock. Those 52 patients were operated preferentially by laparotomy (38.3% versus 12.2%, *p*=0.001). Similarly, patients with a Boey score ≥2 were operated preferentially by laparotomy (35% versus 20.4%, *p*=0.062).

The majority of the patients (*n* = 102; 60.4%) were operated in less than 24 hours after the onset of the symptoms. No differences were found between the two groups regarding the symptoms-to-surgery time interval (*p*=0.700).

Gastric PPU was observed in 80 (47.3%) patients, pyloric PPU in 63 (37.3%) patients, and duodenal PPU in 25 (14.8%) patients. One patient (0.6%) had PPU on a previous gastrojejunal anastomosis.

A nonresection procedure (suture ± omental or round ligament patch) was performed in 164 (97%) patients. There were 5 patients submitted to gastric resection; all of them performed by open surgery: 4 atypical gastrectomies and 1 distal gastrectomy with gastrojejunal anastomosis (Billroth 2).

Regarding only gastric location, biopsy of the ulcer wall was performed intraoperatively in 56 (70%) patients: 8 (36.4%) patients in the laparoscopy group and 48 (82.8%) patients in the laparotomy group, *p* < 0.001. All patients presented no signs of malignancy.

The median operative time in the laparotomy group and in the laparoscopy group was 80 and 100 minutes, respectively (*p*=0.01).

Early complications (<30 days) were found in 59 (34.9%) patients: 9 (18.4%) in the laparoscopy group and 50 (41.7%) in the laparotomy group, *p*=0.004. Regarding the Clavien-Dindo classification, grade I/II complications (mainly respiratory or wound infections treated with antibiotic therapy) were observed in 16 (27.1%) patients, grade III complications were recorded in 12 (20.7%) patients, and grade IV complications were present in 13 (22.0%) patients. Overall mortality was 10.7% (18 patients): 1 (2%) patients in the laparoscopic group and 17 (14.2%) in the laparotomy group, *p*=0.02. Sepsis with multiorganic failure was the most frequent cause of death.

Reoperation was needed in 16 patients: 3 (6.1%) in the laparoscopy group and 13 (10.8%) in the laparotomy group (*p*=0.343). There were 7 cases of ulcer suture dehiscence: 2 (4.1%) in the laparoscopic group and 5 (4.2%) in the open surgery group (*p*=0.980). Suture dehiscence led to surgical reintervention in 6 patients and one patient was treated nonoperatively with antibiotics and percutaneous drainage. All reoperations were performed by midline laparotomy. Other causes of reoperation were tertiary peritonitis (5 patients), evisceration (3 patients), iatrogenic lesion of the spleen (one patient), and ulcer relapse (one patient).

Laparoscopy was associated with a median length of hospital stay of 6 days (4–79) compared to 7 days (1–152) in the laparotomy group, *p*=0.001. The median time for resumption of oral intake after surgery was 3 days in the laparoscopy group compared to 4 days in the laparotomy group, *p*=0.021.

Late complications (>30 days) were present in 5 (10.4%) patients submitted to laparoscopy repair of PPU and in 15 (16%) patients who underwent open surgery (*p*=0.369). The majority (14/20) of these complications were incisional hernias.

Despite any selection bias, laparoscopy reduced the probability of postoperative complications and mortality in 68.5% (crude OR 0.315; CI 95% 0.140–0.707, *p*=0.005) and 87.4% (crude OR 0.126; CI 95% 0.016–0.967, *p*=0.047), respectively.

Female gender (crude OR 2.378; CI 95% 1.175–4.812, *p*=0.016) and age (crude OR 1.057; CI 95% 1.034–1.081, *p* < 0.001) were risk factors for postoperative early complications and mortality (crude OR 4.301; CI 95% 1.574–11.752, *p*=0.004, and crude OR 1.047; CI 95% 1.016–1.079, *p*=0.003, resp.). Therefore, when laparoscopy was adjusted for gender and age, no significance was reached regarding postoperatory complications (*p*=0.083) or mortality (*p*=0.133).

### 3.1. Sepsis Subgroup Analysis

In the group of patients with sepsis (*n* = 52), 46 (88.5%) patients were submitted to open repair of PPU. In these subgroups of patients, significant differences were observed in postoperative complications (33.3% in the laparoscopy group and 76.1% in the laparotomy group, *p*=0.05). Laparoscopy was associated with a decrease of 84.3% in postoperative complications (crude OR 0.157; CI 95% 0.025–0.977, *p*=0.047). Gender and age were not associated with postoperative complications in this subgroup. However, when the laparoscopy group was adjusted for these 2 variables, no significance was reached regarding postoperative complications (*p*=0.064). No other differences between the laparoscopy and the laparotomy group were found: results are summarized in Table 2.

## 4. Discussion

In this series of patients, PPU occurred more frequently in male patients and within a median age of 52 years, in line with historical cohorts. The trend observed in recent occidental reports [3, 9] of equal gender distribution and older age was not confirmed in this specific population. However, like these and other recent papers reported, PPU is now more frequent in the stomach than in the duodenum [3, 4, 6, 9].

Since PPU is a serious complication of PUD, the symptoms-to-surgery interval is an important prognostic factor and is related to increased morbidity and mortality [11–13]. Every hour of delay may reduce the probability of survival by 2–4% [12]. The majority of patients in this series were operated in the first 24 hours.

Risk stratification was performed using the Boey score, one of the most specific validated scores used for PPU [11, 14]. Furthermore, this study used the new Sepsis-3 criteria to assess the severity and to predict outcomes of patients with PPU: to our knowledge, these criteria have never been used before in patients with PPU.

According to Sepsis-3 criteria, in this series, patients with sepsis were operated preferentially by laparotomy and had higher rates of postoperative complications and mortality. Although a Boey score ≥2 is referred to be a marker of poor prognosis, in this series, only 34 of the 52 patients with Boey score ≥2 presented sepsis at the same time. Patients with Boey score ≥2 were not significantly associated with laparotomy repair of PPU or higher rates of complications. Consequently, in our series, Boey score was less accurate than the Sepsis-3 criteria to predict patients' outcomes.

According to the recent guidelines from the World Society of Emergency Surgery (WSES), a laparoscopic approach in PPU is recommended in “stable” patients, while an open approach should be performed in the absence of laparoscopic skills and equipment as well in “unstable” patients [15]. In our series, laparoscopic repair of PPU was performed essentially in patients with a better prognosis (patients with no sepsis). Interestingly, we observed that sepsis at admission was more frequent in women than in men. So, when comparing the laparoscopic group with the open one, the male : female ratio was higher in the laparoscopic group.

This study has some limitations. The patients were not randomized to laparotomy or laparoscopic treatment of PPU. The surgical approach was decided case by case and taking into account the surgeons' expertise in laparoscopy. We observed that laparoscopic repair of PPU was accomplished in 29% of patients which is in line with recently published data reporting that laparoscopic repair is used in 3–33% of patients with PPU [6, 8, 9].

The conversion rate of this series (18,3%) is lower than other reports (25–44%) [8, 9, 16, 17]. Mean operative time was longer in the laparoscopic group, as observed in these recent papers.

Biopsy of the ulcer wall was done less frequently when the treatment was performed by laparoscopy. We assume that intraoperative adverse conditions were the main reason for this difference. Although controversial, we may consider that surgical repair of PPU is a life-saving surgery often performed in a “damage-control” setting and definitive diagnosis may be postponed for postoperative endoscopic biopsies of nonhealing ulcers. This strategy may delay diagnosis. However, it is an acceptable strategy in selected cases with no effects on prognosis, especially if we take into account the fact that the optimal oncologic treatment of a perforated gastric cancer in the acute setting is difficult to achieve. As malignancy was considered as an exclusion criterion, we could not analyse such differences and few data are available regarding this topic.

PPU is a serious complication of PUD with mortality and mobility that can reach 30% and 50%, respectively [2, 5]. Postoperative early complications occurred in 59 (34.9%) of our patients with an overall mortality of 10.7%. When analysed by groups, the laparoscopic group had a significantly lower rate of complications and mortality compared to the laparotomy group. However, there was a selection bias of patients with better prognosis (younger, males, and without sepsis criteria). When the analysis was adjusted for sex and age, statistically significant differences were not found regarding complications or mortality. Accordingly, other studies reported no significant differences between laparoscopy and laparotomy regarding postoperative complications or mortality [8, 17, 18]. However, a shorter hospital stay is observed for patients submitted to laparoscopic repair of PPU, as already reported [6, 17].

When sepsis was present, there were few patients treated by laparoscopy. Nonetheless, we observed that outcomes after laparoscopic repair are noninferior to the outcomes of open repair of PPU, even in the presence of severity criteria. We expect that, as surgical expertise and perioperative care continuously improve, we may observe in the near future the full spectrum of advantages attributed to the minimally invasive techniques, as described for other procedures.

## 5. Conclusion

Recent guidelines started to suggest which kind of patients could benefit from a laparoscopic approach instead of a classic open one for the treatment of PPU. However, the selection criteria for a correct assignment are not very clear yet. Specific criteria to identify a “stable” or “unstable” patient like WSES are required. Sepsis-3 criteria proved to be an accurate score in predicting outcomes for PPU and maybe they could be used for this purpose. In our opinion, patients without sepsis criteria benefit from minimally invasive approaches. The presence of sepsis or septic shock may not be considered an absolute contraindication for laparoscopic repair of PPU but additional studies are required to assess feasibility and safety outcomes in this subset of patients.

## Figures and Tables

**Table 1:** Surgical approaches.

Variable	Laparoscopy *n* = 49 (29%)	Laparotomy *n* = 120 (71%)	Total *n* = 169 (100%)	*p* value
*Sex*				**0.009**
Male	43 (87.8%)	82 (68.3%)	125 (74%)	
Female	6 (12.2%)	38 (31.7%)	44 (26%)	
Age (years, median, and range)	48 (21–81)	53 (21–97)	52 (21–97)	**0.002**
Comorbidities	25 (51%)	72 (60%)	97 (57.4%)	0.284
Sepsis criteria	6 (12.2%)	46 (38.3%)	52 (30.8%)	**0.001**

*Sepsis group*				**0.003**
Nonsepsis	43 (87.8%)	74 (61.7%)	117 (69.2%)	
Sepsis	5 (10.2%)	28 (23.3%)	33 (19.5%)	
Septic shock	1 (2%)	18 (15.0%)	19 (11.2%)	

*Boey score*				0.317
0	22 (44.9%)	43 (35.8%)	65 (38.5%)	
1	17 (34.7%)	35 (29.2%)	52 (30.8%)	
2	7 (14.3%)	28 (23.3%)	35 (20.7%)	
3	3 (6.1%)	14 (11.7%)	17 (10.1%)	

*Categorical Boey score*				0.062
Boey score <2	39 (79.6%)	78 (65%)	117 (69.2%)	
Boey score ≥2	10 (20.4%)	42 (35%)	52 (30.8%)	

*Symptoms-surgery delay (hours)*				0.700
<12	19 (38.8%)	41 (34.2%)	60 (35.5%)	
12 < 24	13 (26.5%)	29 (24.2%)	42 (24.9%)	
>24	17 (34.7%)	50 (41.7%)	67 (39.6%)	

*Ulcer localization*				0.305
Gastric	22 (44.9%)	58 (48.3%)	80 (47.3%)	
Pyloric	16 (32.7%)	47 (39.2%)	63 (37.3%)	
Duodenal	11 (22.4%)	14 (11.7%)	25 (14.8%)	
Gastrojejunal anastomosis	0 (0%)	1 (0.8%)	1 (0.6%)	

*Procedure*				0.323
Suture (nonresection procedure)	49 (100%)	115 (95.8%)	164 (97%)	
Resection procedure	0 (0%)	5 (4.2%)	5 (3%)	

*Biopsy (only gastric location)*				**<0.001**
Yes	8 (36.4%)	48 (82.8%)	56 (70%)	
No	14 (63.6%)	6 (10.3%)	20 (25%)	
Not applied	0 (0%)	4 (6.9%)	4 (5%)	
Operative time (minutes, median, and range)	100 (40–188)	80 (40–260)	90 (40–260)	**0.01**
Early complications (<30 days)	9 (18.4%)	50 (41.7%)	59 (34.9%)	**0.004**

*Complications (Clavien-Dindo)*				**0.032**
Grade I	0 (0%)	1 (2%)	1 (1.7%)	
Grade II	3 (33.3%)	12 (24%)	15 (25.4%)	
Grade III	5 (55.6%)	7 (14%)	12 (20.3%)	
Grade IV	0 (0%)	13 (26%)	13 (22%)	
Grade V	1 (11.1%)	17 (34%)	18 (30.5%)	
Mortality	1 (2.0%)	17 (14.2%)	18 (10.7%)	**0.02**
Suture dehiscence	2 (4.1%)	5 (4.2%)	7 (4.1%)	0.980
Reoperation	3 (6.1%)	13 (10.8%)	16 (9.5%)	0.343
Late complications (>30 days)	5 (10.4%)	15 (16%)	20 (14.1%)	0.369
Hospital stay (days, median, and range)	6 (4–79)	7 (1–152)	7 (1–152)	**0.001**
Oral intake (days, median, and range)	3 (1–7)	4 (2–42)	3.50 (1–52)	**0.021**

**Table 2:** Surgical approaches, sepsis group (*n* = 52).

Variable	Laparoscopy *n* = 6 (11.5%)	Laparotomy *n* = 46 (88.5%)	Total *n* = 52 (100%)	*p* value
*Sex*				0.243
Male	5 (83.3%)	27 (58.7%)	32 (61.5%)	
Female	1 (16.7%)	19 (41.3%)	20 (38.5%)	
Age (years, median, and range)	48 (41–78)	68 (39–94)	62.5 (39–94)	0.078
Comorbidities	5 (83.3%)	39 (84.8%)	44 (84.6%)	0.926

Boey score				0.132
0	1 (16.9%)	1 (2.2%)	2 (3.8%)	
1	0 (0%)	16 (34.8%)	16 (30.8%)	
2	2 (33.3%)	15 (32.6%)	17 (32.7%)	
3	3 (50%)	14 (30.4%)	17 (32.7%)	

*Categorical Boey score*				0.651
Boey score <2	1 (16.7%)	17 (37%)	18 (34.6%)	
Boey score ≥ 2	5 (83.3%)	29 (63%)	34 (65.4%)	

*Symptoms-surgery delay (hours)*				0.382
<12	2 (33.3%)	7 (15.2%)	9 (17.3%)	
12 < 24	0 (0%)	7 (15.2%)	7 (13.5%)	
>24	4 (66.7%)	32 (69.6%)	36 (69.2%)	

*Procedure*				0.519
Suture (nonresection procedure)	6 (100%)	43 (93.5%)	49 (94.2%)	
Resection procedure	0 (0%)	3 (6.5%)	3 (5.8%)	

*Biopsy (only gastric location)*				0.848
Yes	1 (100%)	21 (75%)	22 (75.9%)	
No	0 (0%)	5 (17.9%)	5 (17.2%)	
Not applied	0 (0%)	2 (7.1%)	2 (6.9%)	
Operative time (minutes, median, and range)	122.5 (60–165)	100 (42–245)	100 (42–245)	0.483
Early complications (<30 days)	2 (33.3%)	35 (76.1%)	37 (71.2%)	**0.047**

Complications (Clavien-Dindo)				0.262
Grade I	0 (0%)	0 (0%)	0 (0%)	
Grade II	0 (0%)	6 (17.1%)	6 (16.2%)	
Grade III	1 (50%)	3 (8.6%)	4 (10.8%)	
Grade IV	0 (0%)	11 (31.4%)	11 (29.7%)	
Grade V	1 (50%)	15 (42.9%)	16 (43.2%)	
Mortality	1 (16.7%)	15 (32.6%)	16 (30.8%)	0.653
Late complications (>30 days)	1 (20%)	9 (32.1%)	10 (30.3%)	0.586
Hospital stay (days, median, and range)	7.5 (5–79)	13.5 (1–152)	13 (1–152)	0.354
Oral intake (days, median, and range)	3 (1–5)	4 (2–42)	4 (1–42)	**0.029**

## Data Availability

The demographic and clinical-pathological characteristics and data related to the surgical procedures and outcomes used to support the findings of this study are included within the article. All data were retrieved from the internal electronic database (“SClinic”).
